# The role of national registries in improving patient safety for hip and knee replacements

**DOI:** 10.1186/s12891-017-1773-0

**Published:** 2017-10-16

**Authors:** Anne Lübbeke, Alan J. Silman, Daniel Prieto-Alhambra, Amanda I. Adler, Christophe Barea, Andrew J. Carr

**Affiliations:** 1 Division of Orthopaedic Surgery and Traumatology, Geneva University Hospitals and Geneva University, Geneva, Switzerland; 2 Nuffield Department of Orthopaedics, Rheumatology and Musculoskeletal Sciences, University of Oxford, Oxford, UK; 3 Wolfson Diabetes and Endocrine Clinic, Institute of Metabolic Science, Addenbrooke’s Hospital, Cambridge University Hospitals NHS Foundation Trust, Cambridge, UK; 4 National Institute for Health and Care Excellence, 10 Spring Gardens, London, UK

## Abstract

**Background:**

The serious adverse events associated with metal on metal hip replacements have highlighted the importance of improving methods for monitoring surgical implants.

The new European Union (EU) device regulation will enforce post-marketing surveillance based on registries among other surveillance tools. Europe has a common regulatory environment, a common market for medical devices, and extensive experience with joint replacement registries. In this context, we elaborate how joint replacement registries, while building on existing structure and data, can better ensure safety and balance risks and benefits.

**Main text:**

Actions to improve registry-based implant surveillance include: enriching baseline and diversifying outcomes data collection; improving methodology to limit bias; speeding-up failure detection by active real-time monitoring; implementing risk-benefit analysis; coordinating collaboration between registries; and translating knowledge gained from the data into clinical decision-making and public health policy.

**Conclusions:**

The changes proposed here will improve patient safety, enforce the application of the new legal EU requirements, augment evidence, improve clinical decision-making, facilitate value-based health-care delivery, and provide up-to-date guidance for public health.

## Background

The need to improve post-marketing surveillance of the safety of implants has been highlighted by serious complications, especially those with a delayed onset, as illustrated by pseudo-tumours developing in patients with metal-on-metal implants [1]. In this piece we consider the future role of registries for joint replacement and what changes will be required to best inform decisions on the safety of both existing and novel hip and knee implants.

### Hip and knee replacement incidence

**Fig. 1:**
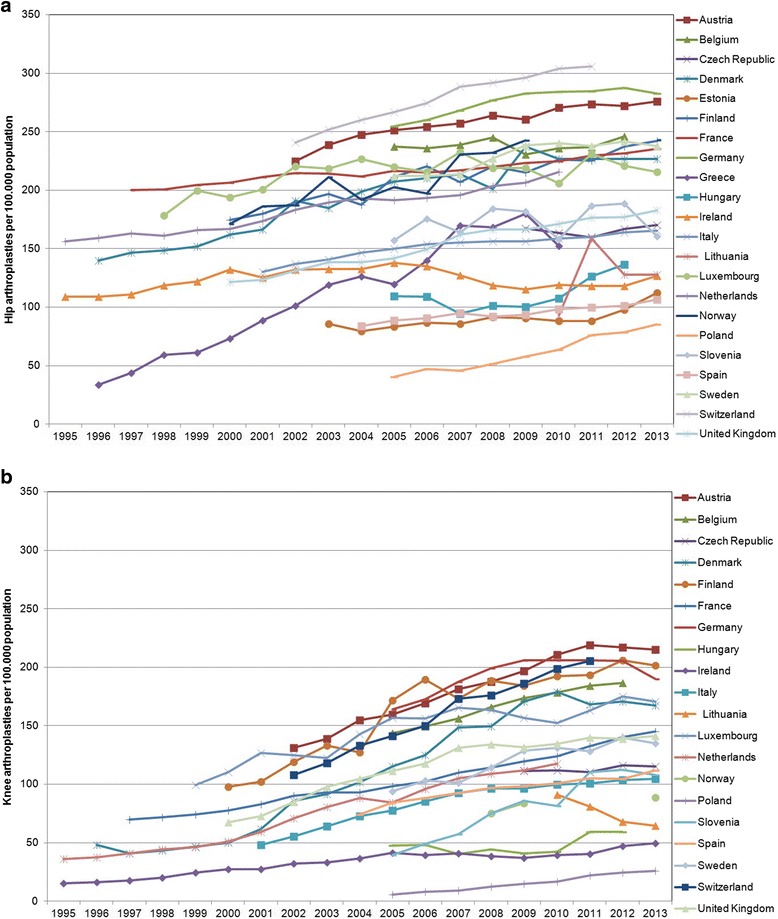
**a**-**b** Data taken from OECD (2016), “Hip and knee replacement”, Health Care utilization, http://stats.oecd.org/index.aspx?DataSetCode=HEALTH_STAT#, (accessed on 8 June 2016). For Norway and Switzerland OECD (2013), Health at a Glance 2013: OECD Indicators, 10.1787/health_glance-2013-en. Permission was obtained from OECD (PACRights@oecd.org) on September 18, 2017

Total hip and knee replacements are very common, and generally considered highly cost-effective orthopaedic procedures [2–4]. In 2005, about 745,000 hip (total and partial) and 430,000 knee replacements were performed in Europe according to the Organisation for Economic Co-Operation and Development (OECD). By 2012, these numbers increased by 10% and 30% respectively to about 820,000 hips and 560,000 knees implanted [5]. Because of rising trends both in obesity prevalence and in life expectancy [6], together with a broadening of the indications for surgery, these numbers have been predicted to increase further [7, 8]. European hip and knee replacement incidence rates from the latest OECD report [5] are shown in Fig. 1a-b.

### Joint replacement registries

Registries of joint replacements have since 1975 pioneered voluntary monitoring of real-world treatment on a national level with a focus on long-term surveillance of implant survival [9]. For the majority of hip and knee replacements implant survival has been substantial and failure leading to revision surgery has remained an infrequent event. Other important potential adverse health consequences that have been evaluated include the short- and long-term risk of death and cancer [10–12]. The relatively low occurrence of complications on the one hand, and the very long-term follow-up required to characterise revision risk on the other hand, make registries well suited for long-term monitoring. Registries have focused on revision as the key outcome for several reasons: (1) it is the major indicator of an implant’s quality; (2) it constitutes a substantial burden to the patient and the society (costs); and (3) it is a “hard endpoint” (reproducible, comparable). A main issue with revision as the sole indicator of success or failure is that revisions can happen long after surgery [13] and as a consequence, not always directly inform the surgeon as to the quality of his/her implant choice. A surgeon’s perceived “performance” of an implant, which influences future implant choice, is likely to also depend on factors such as quality and length of the patient’s recovery, and the ease of surgery and postoperative period, neither of which registries currently capture. Another important issue with revision as the sole indicator is that not all failures are revised highlighting the importance of other measures of failure and success such as patient-reported outcomes (PROMs) and satisfaction [14, 15].

### Implant vs. drug surveillance

Compared to drug safety monitoring, implant surveillance is characterised by several substantive differences: first, new implants do not need pivotal randomised clinical trials before they are licensed for use; second, the implanted material remains present for decades and so adverse consequences can arise in the longer term making their detection challenging [13, 16, 17]; third, the surgeon’s experience and learning curve based on the implants’ level of complexity are unique to device evaluation and important co-determinants of outcome [18]; fourth, implants frequently undergo incremental changes [18]; and fifth, a large number of different implants are available on the market for a limited number of clinical indications (ie several ‘in class’ marketed very closely in time compared to normally a single novel pharmaceutical) [19, 20].

### Metal-on-metal failure

Much higher failure rates have been observed after metal-on-metal hip replacement as compared to other bearing surfaces [21, 22], and a substantial number of patients have already endured revision surgery. Another large group of so far unrevised patients is still at risk for local and/or systematic adverse effects of metal debris [23]. Despite the existence of joint replacement registries the hazards of metal-on-metal hip replacement were not identified sufficiently early to protect public health. Major reasons for this included, among others [24], the prime focus on only one adverse outcome (revision), lack of “real-time” detection of adverse events, limited availability of comparative studies [24], no widely implemented guidance on what is considered acceptable safety and effectiveness, and sparse information on patient characteristics. It has been widely agreed [2, 19, 25–27] for some time [28–30] that the processes for post-marketing surveillance of joint replacements are insufficient. But the inadequate evaluation of the widely available metal-on-metal hip implants has revealed deficiencies in device safety that resulted in major public health concerns [31, 32] and finally prompted the European Union (EU) to revise device regulation.

### New EU medical device regulation

The new EU medical device regulation adopted in April 5, 2017 [33] and applicable for medical devices after a transition period of three years, includes measures to strengthen both pre- and post-marketing surveillance. Key elements are: “a new pre-market scrutiny mechanism with the involvement of a pool of experts at EU level; reinforcement of the criteria for designation and processes for oversight of Notified Bodies; improved transparency through the establishment of a comprehensive EU database on medical devices and of a device traceability system based on Unique Device Identification; the introduction of an “implant card” containing information about implanted medical devices for a patient; the reinforcement of the rules on clinical evidence, including an EU-wide coordinated procedure for authorisation of multi-centre clinical investigations; the strengthening of post-market surveillance requirements for manufacturers; improved coordination mechanisms between EU countries in the fields of vigilance and market surveillance” [34]. The new regulation will rely on registries for post-marketing surveillance among other surveillance tools [33, 35, 36]. Registry surveillance could start from “first-in-human experience onward” as recently suggested in a framework for evaluating and regulating medical device use [37].

Against this background, we believe that joint replacement registries, while building on existing structure and data, can better ensure safety and balance risks and benefits. We describe what changes registries need in terms of gathering data, linkage to other data sources, and approach to analysis, and we discuss the value of multiple registries working together.

## Main text

### Enriching data from registries, and improving analyses

Currently most registries provide detailed information on implants and surgery, but have little information on patient characteristics and outcomes other than implant revision. Registries must collect this additional information, which will then permit investigators (1) to analyse additional indicators of success and failure other than revision (e.g. PROMs) as well as surrogates of failure (e.g. early abnormal radiographic findings [38]), (2) to adjust for potential confounders when comparing treatments, (3) to evaluate causal mechanisms, and (4) to develop a personalised approach to treatment. Thus, registries should capture patient co-morbidities and health behaviours such as smoking and obesity, which could confound or modify the risks of adverse events [39]. Although the most robust and complete data need to be gathered prospectively as part of the primary data collection of the joint registries, reduced data completeness and accuracy may jeopardize this goal. In practice there is currently greater reliance on linkage to secondary data sources to obtain additional data. Secondary sources include primary and secondary care data and data from registries such as for mortality, cancer or drugs. For example, researchers in the United Kingdom recently linked the National Joint Registry to the Clinical Practice Research Datalink to study safety issues related to the use of metal-on-metal implants [40], and also to the Hospital Episode Statistics (inpatient records) to compare uni-compartmental versus total knee replacement [41]. In the latter case, the more detailed information about the patient’s characteristics at the time of surgery and about reoperations and readmissions - obtained from the inpatient records - increased the number of outcomes evaluated as well as the ability to adjust for differences between the two treatment groups. Another example is the linkage to databases that record medications (e.g. prolonged antibiotics or pain medication use), which has been shown to offer a useful surrogate measure for prosthesis infection [42] and revision [43]. In addition, linking to a system designed for spontaneously reporting adverse events [44] may have the potential to improve the detection of failures. Finally, integrating health economics data within registries via primary data collection and/or linkage to secondary data may improve clinical and public health decision-making.

Improving data analysis requires speeding up failure detection and bias minimisation including, but not limited to, confounding by indication. First, stakeholders including manufacturers need to adopt strategies to improve post-authorisation safety surveillance, which are increasingly used to detect adverse events in vaccine and drug surveillance [45, 46] and have become routine when assessing drug post-marketing (EU Regulation No 1027/2012). Regulators should prioritise real-time monitoring of devices by analysing specific risks [47]. Secondly, researchers, regulators and manufacturers should systematically use measures of benefit and risk [48, 49] including PROMs of pain, function and activity, health-related quality of life, satisfaction, and costs to compare devices from a societal and policy-makers’ perspective. Third, when possible randomized trials should be nested in registries. This has the potential to combine the advantages of both study designs and to facilitate the conduct of multi-centre trials with reduced duration and costs [50, 51]. Fourth, researchers should test and incorporate methods (e.g. propensity score methods, sequential cohort analyses among others) developed to reduce bias and confounding when evaluating drugs and vaccines in observational studies [52–55]. Fifth, there is a need to stratify the risk of implant failure and other adverse events by factors specific to patients, surgery, implant, and environment. This may allow stakeholders to target improvements to subgroups, and to inform case-mix adjustment models. Finally, methods for data analyses at an aggregate level should be applied to estimate the comparative effectiveness of multiple treatments [56].

### Maximising the value from multiple (national) registries

Europe has a common regulatory environment and a common market for medical devices. It also has extensive experience with joint replacement registries (e.g. Scandinavian countries, United Kingdom). Over the last 15 years registries have expanded to many other parts of Europe creating the opportunity to harmonise [57, 58] and extend data collection (e.g. International Society of Arthroplasty Registries (ISAR), Network of Orthopaedic Registries of Europe (NORE)) and to engage in multinational initiatives (Nordic Arthroplasty Register Association (NARA)) [59, 60]. These efforts constitute a basis for a coordinated European-wide evaluation of outcomes, which will provide:

## Greater variation of implants, populations, and environment

The variety of implants used in Europe is large, and varies by country. For example 67% of the total hip replacements recorded in the Swedish Hip Arthroplasty Register are cemented compared to 36% in the National Joint Registry for England, Wales, Northern Ireland and the Isle of Man and 15% in the Danish Hip Arthroplasty Registry according to the most recent annual report. This between-country variation constitutes a natural experiment [52], which enables one to compare devices under the condition of ‘quasi-randomisation’ assuming that the data are accurate, harmonised and sufficiently detailed to adjust for baseline imbalances. A multinational initiative would provide the coordination, infrastructure and methodology necessary to evaluate international differences, which would be difficult to achieve in a randomized trial. This between-registry evaluation has already successfully been established in the Scandinavian countries through the NARA collaboration [59, 61].

## Higher volume and reduced time to discover poor implants

Combining the data from the existing registries will increase the numbers of an implant or surgical technique possible to evaluate within a fixed time span. This is critical for newly marketed prostheses, as they will be available only in small numbers in each registry. Pooling results would permit regulators to identify safety issues earlier and to decrease disability and costs associated with failures. Combining data will also allow testing the consistency of the findings by validating them in different populations and settings [62]. Finally, an increased sample size will increase the precision of effect estimates and provide power for stratified analyses.

### Beyond registries

Translating knowledge gained from the data into public health policy and health care delivery [63, 64] will be as important as changing the EU device legislation and improving future orthopaedic surgeon education [27]. Methods for stepwise introduction of new implants [65, 66] and new benchmark revision rates [67, 68] have also been proposed. Notwithstanding the challenges involved when using observational data for such evaluations, integrating quality and health outcomes from registries into health technology appraisals will undoubtedly improve them.

## Conclusions

Richer sources of data, improved information technology and changing regulatory environment provide new opportunities to introduce safe orthopaedic implants, but numerous challenges remain. They relate to the use of observational data (especially issues with systematic error), to appropriate comparator identification and risk adjustment, to concerns with data quality, safety and privacy, and to issues of practicability, such as merging aggregate data from diverse sources, identifying signals and surrogates for clinically relevant adverse events, and measuring care processes. Finally, managing health policy and legal implications related to benchmarking and outlier identification as well as reconciling international and national priorities will be important.

The current infrastructure surrounding registries for joint replacement has improved but has not, as yet, solved all the problems with the safety of joint implants as demonstrated by metal-on-metal hip devices. Suggested actions to improve registry-based implant surveillance include enriching baseline and diversify outcomes data collection, improving methodology to limit bias, speeding-up failure detection by active real-time monitoring, implementing risk-benefit analysis, coordinating collaboration between registries, and translating knowledge gained from the data into clinical decision-making and public health policy. These changes will improve patient safety, enforce the application of the new legal EU requirements, augment evidence, improve clinical decision-making, facilitate value-based health-care delivery, and provide up-to-date guidance for public health.
